# Development of a Chemogenetic Approach to Manipulate Intracellular pH

**DOI:** 10.1021/jacs.3c00703

**Published:** 2023-05-24

**Authors:** Asal Ghaffari Zaki, Seyed Mohammad Miri, Şeyma Çimen, Tuba Akgül Çağlar, Esra N. Yiğit, Mehmet Ş. Aydın, Gürkan Öztürk, Emrah Eroglu

**Affiliations:** † Regenerative and Restorative Medicine Research Center (REMER), Research Institute for Health Sciences and Technologies (SABITA), Istanbul Medipol University, Istanbul 34810, Türkiye; ‡ Molecular Biology, Genetics and Bioengineering Program, Faculty of Engineering and Natural Sciences, Sabanci University, Istanbul 34956, Türkiye; § Department of Physiology, International School of Medicine, Istanbul Medipol University; Istanbul 34810, Türkiye

## Abstract

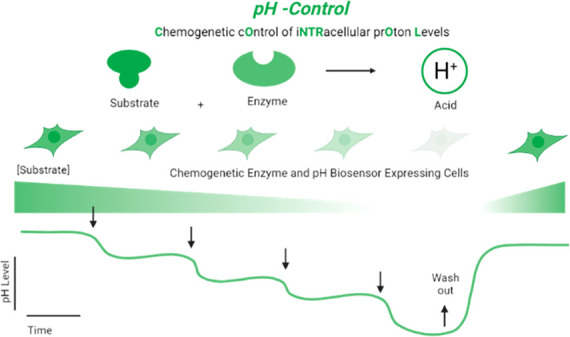

Chemogenetic Operation of iNTRacellular prOton Levels (pH-Control) is a novel substrate-based enzymatic method that enables precise spatiotemporal control of ultralocal acidification in cultured cell lines and primary neurons. The genetically encoded biosensor SypHer3s showed that pH-Control effectively acidifies cytosolic, mitochondrial, and nuclear pH exclusively in the presence of β-chloro-d-alanine in living cells in a concentration-dependent manner. The pH-Control approach is promising for investigating the ultralocal pH imbalance associated with many diseases.

Intracellular pH levels are tightly regulated.^1^ Gene expression, cell motility, and metabolic processes are a few examples of the many cellular processes under the control of local pH fluctuations.^2^ Hence, multiple disorders, such as cancer,^3^ cardiovascular diseases,^4^ and neurological diseases,^5^ may be associated with the dysregulation of pH. The ability to monitor^6^ and manipulate^7^ intracellular pH levels directly inside a single cell has enormous ramifications for understanding subcellular and suborganelle processes, disease diagnosis, and developing novel therapeutic strategies.^8,9^ Several technologies have been advanced to investigate the role of pH at single cell level; however, conventional methods such as the application of micropipettes,^10^ genetic or chemical manipulation of proton pumps,^11^ optogenetic approaches,^12^ and small chemical inhibitors^13^ have off-target effects or are less practical (Supporting Information Figure S1). Therefore, the lack of tractable experimental tools permitting manipulating pH levels with high spatiotemporal resolution in the acidic range undermines studying the relationship between pH imbalance and cell function in health and disease.

We present pH-Control, an acronym for Chemogenetic Operation of iNTRacellular prOton Levels, as a novel chemogenetic approach that we have combined with the genetically encoded biosensor SypHer3s^14^ for simultaneous visualization of ultralocal acidification in living cells (Figure 1a). Substrate-based chemogenetic tools are silent recombinant proteins until their biochemical stimulus - typically an unnatural amino acid is provided.^15^ Combined with genetically encodable biosensors, these experimental systems have opened up new lines of investigation, allowing the analysis of intracellular pathways that modulate physiological and pathological cell responses.^16^

**Figure 1:**
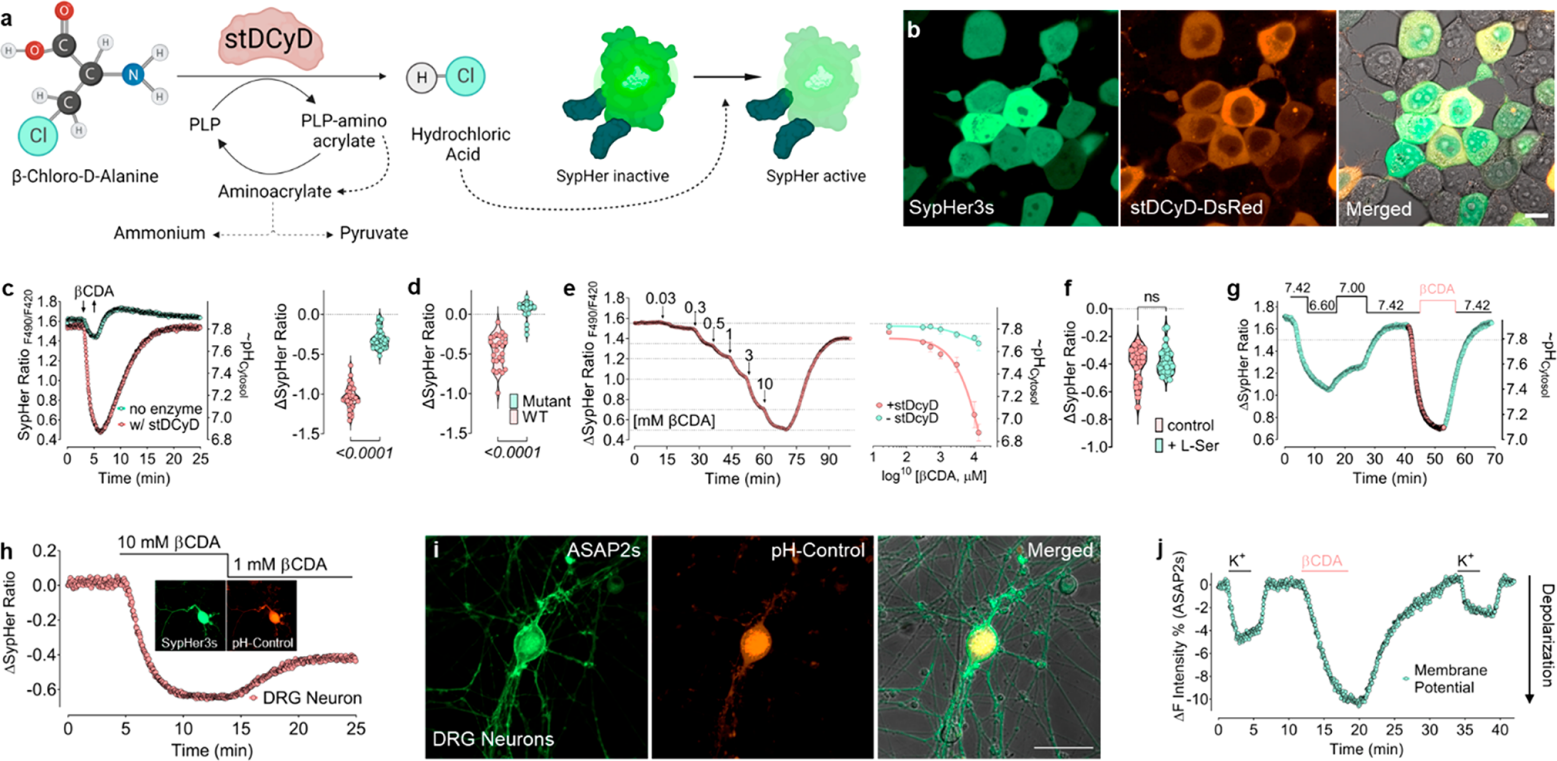
Characterization of pH-Control. (a) Schematic representation of the pH-Control pathway and simultaneous visualization with the pH-sensitive biosensor SypHer3s. (b) Representative confocal images of HEK293T cells coexpressing DsRed-stDCyD and SypHer3s. Scale bar = 20 μm. (c) Real-time SypHer3s traces of cytosolic pH in WT cells (*n* = 3/39) or cells expressing DsRed-stDCyD (*n* = 3/32) in response to 13.4 mM βCDA. (d) Violine plot shows SypHer3s biosensor responses in cells expressing the WT DsRed-stDCyD (*n* = 4/21) and mutated and nonfunctional DsRed-stDCyD (*n* = 4/18) upon administration of 1 mM βCDA. (e) The left panel shows a representative curve of SypHer3s in response to various concentrations of βCDA, as indicated in the figure. The right panel shows a concentration–response curve in HEK293T cells without enzyme (green curve) or expressing stDCyD (red curve) upon administration of indicated concentrations of βCDA. *N* = 3 for all experiments and *n* = 8–49 individual cells. (f) The violin plot shows the competitive inhibitory effect of l-serine in HEK293T cells expressing DsRed-stDCyD-NES and SypHer3s-NES. Treated cells were preincubated for 1 h with 1 mM l-Serine before the imaging experiment (*n* = 3/35). The control group was incubated in a storage buffer (*n* = 3/27); Student’s *t* test has been applied. (g) Representative real-time traces of HEK293T cells coexpressing SypHer3s and pH-Control in response to imaging medium with different pH levels and 1 mM βCDA as indicated (*n* = 3/17). (h) Representative real-time traces of SypHer3s signals in DRG neurons expressing pH-Control in response to 10 or 1 mM βCDA. The inset shows representative confocal images of DRG neurons coexpressing SypHer3s and pH-Control. (i) Representative confocal images of dorsal root ganglion neurons coexpressing ASAP 2s and pH-Control 8 days after viral infection. Scale bar = 50 μm. (j) Representative real-time curve shows signals of the voltage sensor ASAP 2s in DRG neurons coexpressing pH-Control in response to high potassium (50 mM) and 10 mM βCDA as indicated (similar results were obtained from 4 different experiments and 11 individual cells). Student’s *t* test was applied.

pH-Control is a chimera of a red fluorescent protein variant (DsRed) and a *Salmonella typhimurium*-derived enzyme termed d-cysteine desulfhydrase (stDCyD).^17^ stDCyD converts the unnatural amino acid β-chloro-d-alanine (βCDA) to its corresponding α-ketoacid and generates the byproducts hydrochloric acid (HCl), ammonium (NH_4_^+^), and pyruvate in the presence of the cofactor pyridoxal 5′ phosphate (PLP). βCDA is a well-established antibacterial agent and cannot be metabolized by human cells and tissues.^18^ The stDCyD enzyme is differentially targetable to subcellular locales where it remains quiescent until its substrate (βCDA) is provided to generate HCl. Theoretical calculations and experimental approaches showed that the amount of generated byproducts is neglectable (Supporting Information Table S1 and Figure S2). At the same time, the change in [H^+^] equals a 900% increase upon a pH change of 1 order of magnitude during the enzymatic activity of stDCyD (Supporting Information Table S1). *In vitro* characterization of the recombinant DsRed-stDCyD using Seahorse XFe96 analyzer showed that the acidification rate of the enzyme increased in a substrate concentration-dependent manner and sustainably altered the pH in the medium oxygen-independently (Supporting Information Figure S3).

Overexpressing pH-Control with SypHer3s in cultured cells (HEK293T) did not show any visible toxicity (Figure 1b) even if differentially targeted to the cytosol, mitochondria, or cell nucleus (Supporting Information Figure S4). Administration of high concentrations of βCDA to cells expressing pH-Control yielded robust intracellular acidification as documented by the pH-sensitive biosensor SypHer3s (Figure 1c). βCDA-induced acidification showed heterogeneous SypHer3s responses. Thus, we sought to investigate the correlation between enzyme expression levels and acidification rate (Supporting Information Figure S5a). Our results highlighted that the variations in acidification are independent of the expression levels of DsRed-stDCyD. They are likely due to the influence of several other factors, including differences in βCDA metabolism, amino acid transportation, and pH buffering variations even in clonal cells. Conversely, our in vitro analysis using purified DsRed-stDCyD showed a positive correlation between enzyme concentration and acidification rate in the medium under constant substrate levels. This observation suggests that the intracellular expression levels of the enzyme are constrained and cannot fluctuate significantly to exert a notable effect (Supporting Information Figure S5b).

Wild-type cells without the enzyme showed marginal response to βCDA (Figure 1c). The insignificant acidification observed in response to high concentrations of βCDA may be attributed to the fact that some amino acid transporters, such as the proton-coupled transporter 1 (PAT1) facilitate the transport of d-alanine using proton symport;^19^ therefore, transport of βCDA in wild-type cells may accompany slight acidifications. Additional investigations can provide further insights into this hypothesis.

A single mutation at position Y287F in the stDCyD enzyme yielded a dysfunctional control construct incapable of acidifying cells upon provision of βCDA (Figure 1d and Supporting Information Figure S6).

Constitutive administration of different levels of βCDA to cells expressing pH-Control showed a concentration-dependent and fully reversible SypHer3s response (Figure 1e, left panel). At the same time, cells only expressing SypHer3s remained unresponsive to the same treatment (Figure 1e, right panel).

To estimate the pH-Control mediated acidification capacity, we calibrated the SypHer3s biosensor *in cellulo* and *in vitro* as recombinant proteins (Supporting Information Figure S7a). We found the highest detection range of the pH biosensor between pH 7.5 and 9.0. Although the p*K*_a_-value of the probe is ∼7.8, our results *in cellulo* demonstrate that the detection range of the biosensor is sufficient to dynamically measure intracellular changes in pH even in response to high levels of βCDA (Supporting Information Figure S7b).

Figure 1g highlights the advantage of using the pH-Control approach; intracellular acidification resulting from reducing the pH levels of the extracellular imaging medium permits only global pH changes yet to a limited degree, as revealed by SypHer3s calibration (Supplementary Note 1). Moreover, this experiment demonstrates that low concentrations of βCDA allow higher degrees of acidification. Our results imply that pH-Control allows manipulation of intracellular pH in 1 order of magnitude, typically from pH ∼7.7 to ∼6.8 in the cytosol (Supporting Information Figure S7b).

Specificity tests unveiled that the enzyme remained agnostic to d-alanine (data not shown) and showed marginal responses to β-chloro-l-alanine (βCLA) in comparison to βCDA (Supporting Information Figure S8). Previous reports^17^ suggested l-Serine as a competitive inhibitor of stDCyD *in vitro*. In contrast, our results showed that cell treatment with this amino acid did not yield any significant drop in stDCyD acidification capacity, making the chemogenetic pH-Control technology suitable for *in cellulo* and *in vivo* experiments (Figure 1f).

Another critical observation was that after the withdrawal of βCDA, the biosensor’s signal overshot the baseline after recovery, indicating a cellular alkalization, which aligns with a recent report^20^ (Supporting Information Figure S9). To tackle this issue further, we visualized the overcorrection in cells in the presence and absence of monensin and nigericin to disentangle controlled proton transport from H^+^ channels (Supporting Information Figure S9). Intracellular pH overcorrection was diminished when cells were permeabilized with monensin and nigericin.

We next attempted to use the pH-Control method in mouse primary dorsal root ganglion (DRG) neurons. Cells displayed high expression levels of both constructs, pH-Control, and SypHer3s, 8 days following viral transduction (Figure 1h inset). Administration of different concentrations of βCDA yielded strong SypHer3s signals in DRG neurons (Figure 1h). We next sought to test whether pH-Control mediated acidification is sufficient to manipulate intact primary neurons. We used the voltage sensor ASAP 2s,^21^ a GFP-based biosensor targeted to the outer cell membrane, assuming that H^+^ generation would cause depolarization in membrane potential (Figure 1i and Supporting Information Figure S10). Provision of βCDA depolarized primary neurons even stronger than high extracellular potassium (Figure 1j and Supporting Information Figure S11). Overall, our results show that the pH-Control method is effective for cytosolic acidification of both cell lines and primary cells with functional consequences.

In conclusion, we developed pH-Control, a novel substrate-based chemogenetic method that enables temporal and precise manipulation of intracellular pH levels. Even in complex cell systems like neurons, pH-Control is easily combinable with any suitable biosensor for simultaneous imaging of intracellular acidification. We anticipate that the introduction of our new method to transgenic animal model systems in the future will make it possible to dynamically modify pH balance in various cells and tissues alongside the ability to identify new therapeutic targets implicated in pathological acidification and physiological pathways.

## Supporting Information Available

Figures S1–S11 and Tables S1 and S2; additional characterization of pH-Control and SypHer3s as purified proteins and in HEK293 cells and primary neurons. Detailed experimental procedure (PDF)

## Abbreviations

DRG: dorsal root ganglion
stDCyD: *Salmonella typhimurium*d-cysteine desulfhydrase
βCDA: β-chloro-d-alanine
βCLA: β-chloro-l-alanine
HCl: hydrochloric acid
PLP: pyridoxal 5′ phosphate
