# Supplementary material for: Development of a Chemogenetic Approach to Manipulate Intracellular pH
Source: J Am Chem Soc. 2023 May 24;145(22):11899–902. doi: 10.1021/jacs.3c00703 (PMC10251507; doi:10.1021/jacs.3c00703)
Supplement: Supplementary file 1 — ja3c00703_si_001.pdf [file ja3c00703_si_001.pdf]

Supporting Information

Title: Development of a chemogenetic approach to manipulate intracellular pH

Asal Ghaffari Zaki[1,2], Seyed Mohammad Miri[1,2], Şeyma Çimen[1], Tuba Akgül Çağlar[1,2], Esra Nur Yiğit[1], Mehmet Şerif Aydın[1], Gürkan Öztürk[1,3,] & Emrah Eroglu[1,2,*]*

[1]Regenerative and Restorative Medicine Research Center (REMER), Research Institute for Health Sciences and Technologies (SABITA), Istanbul Medipol University, Istanbul 34810, Türkiye
[2]Molecular Biology, Genetics and Bioengineering Program, Faculty of Engineering and Natural Sciences, Sabanci University, Istanbul 34956, Türkiye
[3]Department of Physiology, International School of Medicine, Istanbul Medipol University; Istanbul 34810, Türkiye

*Correspondence: emrah.eroglu@medipol.edu.tr or gozturk@medipol.edu.tr

Methods

Construct Cloning

The cDNA encoding for stDCyD was synthesized commercially (by Twist Bioscience, USA) and subcloned into a CMV driven backbone termed pTwist CMV BetaGlobin, which was further used as a template for all following cloning steps. The primers and template plasmids are listed in table S2. All constructs were cloned into pLenti-MP2 (Addgene plasmid #36097). Cloning fragments such as targeting sequences (mito, NES, and NLS) were PCR amplified with either Taq DNA polymerase (NEB, M0273) or Q5 high-fidelity DNA polymerase (NEB, M0491S) from templates as listed in table S2. The PCR products and cloning vectors were double digested with relevant restriction enzymes (all purchased from NEB), purified from agarose gel, and ligated using T4 DNA ligase (NEB, M0202T). The ligation products were then transformed into NEB stable competent E. coli (C3040H, NEB) cells. The Y287F stDCyD mutant construct was made through overlapping PCR. First, the overlapping fragments were PCR amplified using SalI- stDCyD -For/stDCyD -Y287F-Rev and ApaI-stop- stDCyD -Rev/ stDCyD -Y287F-for primer sets. The PCR products were then used as co-templates for the next round of amplification using SalI- stDCyDFor/Apa1-Stop-stDCyD-Rev primers. The final product was cloned in pLenti-MP2 as previously described. All constructs were verified by whole plasmid sequencing. To create bacterial expression vectors for protein expression and purification purposes, DsRed stDCyD-NES and SypHer-NES were cloned between BamHI and KpnI sites of EKAR2G_design1_mTFP_wt_Venus_wt plasmid (Addgene: cpFRET - FRET based biosensors Kit (Kit #1000000021)) using regular double-digestion and ligation method.

Buffers and Chemicals

Dulbecco's modified Eagle's medium (DMEM), phenol-free DMEM, penicillin and streptomycin, trypsin, and fetal bovine serum (FBS) were purchased from Pan Biotech (Aidenbach, Germany). Transfection reagent Polyjet was purchased from Signagen (Maryland, USA). β-Chloro-D-alanine was purchased from Biosynth Ltd (Compton, United Kingdom). β-Chloro-L-alanine was purchased from Medchem (Istanbul, Türkiye). All chemicals were purchased from NeoFroxx (Einhausen, Germany) unless otherwise stated. Cells outside the CO_2 incubation chamber were maintained in a storage buffer containing 2 mM $CaCl_2$, 5 mM KCl, 138 mM NaCl, 1 mM $MgCl_2$, 1 mM HEPES (Pan-Biotech, Aidenbach, Germany), 0.44 mM KH_2PO_4, 2.6 mM $NaHCO_3$, 0.34 mM NaH_2PO_4, 10 mM D-Glucose, 0.1% MEM Vitamins (Pan-Biotech, Aidenbach, Germany), 0.2% essential amino acids (Pan-Biotech, Aidenbach, Germany), 100 µg/mL Penicillin (Pan-Biotech, Aidenbach, Germany), and 100 U/mL Streptomycin (Pan-Biotech, Aidenbach, Germany). The pH was adjusted to 7.43 using 1 M NaOH. The cell storage buffer was sterilized using a 0.45 µm medium filter (Isolab, Germany).

For live-cell imaging experiments, a HEPES-buffered physiological solution was used consisting of 2 mM $CaCl_2$, 5 mM KCl, 138 mM NaCl, 1 mM $MgCl_2$, 10 mM HEPES, 10 mM D-Glucose, and pH was adjusted to 7.43 using 1 M NaOH.

For SypHer3s calibration experiments in cells with digitonin (D141, Sigma), nigericin (N7143, Merck) and monensin (M5273, Merck) imaging buffered contained 5 mM KCl, 138 mM NaCl, 1 mM $MgCl_2$, 20

mM (HEPES for pH 5.5, 6.0, 6.5, 7.0, and 7.5 or Tris for pH 8.0, 8.5, 9.0, 9.5, 10.0) 10 mM D-Glucose, 0.2 mM EGTA and pH was adjusted to the desired value using either 1 M NaOH or 1M HCl. Cells were pretreated in the buffer containing 5 µM digitonin, 0.7 µM nigericin and 0.7 µM monensin for 12 minutes prior to imaging and then images were taken. To prepare β-Chloro-D-alanine solutions, the powder was dissolved in imaging buffer and the pH was readjusted to 7.43.

Seahorse XFe96 Analyzer Experiments

The Seahorse XFe96 Extracellular Flux Analyzer (Agilent Technologies) was used to monitor extracellular acidification rate (ECAR, -△pH/△t), changes in pH levels, and oxygen consumption rate (OCR, -△O_2/△t) simultaneously in media containing recombinant DsRed-stDCyD. The day before measurement, a sensor cartridge (Agilent Technologies) was hydrated with distilled water and kept in a non-CO_2 incubator overnight. The following day, the cartridge was placed in Seahorse XF calibrant solution for at least 3 hours before the assay. Different concentrations of recombinant DsRed-stDCyD (10, 30, 100, 300, and 1000 nM) and βCDA (0.1, 0.3, 1, 3, and 10 mM) were prepared with pH-adjusted Seahorse XF Base DMEM Media (Agilent Technologies). Varying doses of βCDA were loaded into port A of the Seahorse XF cartridge. Recombinant DsRed-stDCyD solutions in 180 µl assay media were meticulously dispensed into the designated wells of the XFe96-well cell culture microplate (Agilent Technologies) in triplicates. Blank wells (assay medium only) were prepared without recombinant protein for subtracting the background oxygen consumption rate (OCR) and extracellular acidification rate (ECAR) during analysis. Before the real-time measurements, the Seahorse XF cartridge was inserted into the Seahorse Analyzer for calibration. After calibration, the cell culture microplate was inserted into the analyzer, and measurements were carried out using mix/wait/measure times of 1/0/3 minutes intervals. Following three cycles of baseline measurements, varying doses of βCDA were injected into the wells, and OCR, ECAR, and pH levels of the wells were measured simultaneously. Analysis of results was performed using Wave 2.6 software (Agilent Technologies).

Protein expression and purification

pTriEx plasmids containing DsRed-stDCyD and SypHer3s were transformed into Rosetta (DE3) Competent Cells. 16 hours after the transformation, one colony from each construct was inoculated in 5mL LB containing carbenicillin and grown for 8 hours. After 8 hours, cultures were transferred to 250mL LB containing carbenicillin and incubated at 37°C until OD reached 0.4-0.6. Then protein expression was induced using 0.5 mM IPTG for each culture. Cultures were kept at 18°C for 16 hours after IPTG addition. Purification of 6xHistidine tagged proteins was done using the Ni-NTA affinity chromatography method as described elsewhere[1].

Calibration of purified SypHer3s using SpectraMax i3 device

In vitro calibration of SypHer3s was performed using SpectraMax i3 Multi-Mode Microplate Reader for fluorescence detection. Buffers within the pH range of 5.5 to 10.0 were prepared in 2 mL tubes and mixed with 100 nM SypHer3s protein. Each buffer was loaded as triplicates into a solid black bottom 96-

well plate. The solution was excited with 430/9 nm and 485/9nm and emission was collected at 535/15nm. SypHer3s ratio was calculated as Ex485/Ex430.

Cell Culture

Characterization studies have been performed in cultured human embryonic kidney cells (HEK293), grown in a high-glucose (4.5 g/L) complete medium including 10% FBS and 100 µg/ml streptomycin and 100 U/ml penicillin in a humidified incubator (37°C, 5% CO_2). 24 hours before transfection, cells were seeded (~3 × 10^5 cells per well) on a 30 mm glass coverslips No.1 (Glaswarenfabrik Karl Knecht Sondheim, Germany). At ~70–80% confluency, cells were co-transfected with a CMV-driven mammalian expression vectors (pLenti-MP2) encoding for differential targeted SypHer3s or DsRed-stDCyD enzymes using PolyJet transfection reagent according to the manufacturer's instructions. All imaging experiments were performed 24 h after transfection. HEK293T cells were cultured up to passage 30.

Primary Neuron Culture: WT C57BL/6 mice were euthanized via cervical dislocation. Bilateral DRGs from all segments were dissected and then put in an ice-cold RPMI 1640 medium (R0883, Gibco). For enzymatic dissociation, ganglia were incubated with 100 U/mL collagenases (C9407, Sigma) in a neural medium containing 2% B27 (17504-044, Gibco), 2 mM Glutamax-I (35050-61, Gibco), 100 U penicillin, and 100 mg streptomycin (15140-122, Gibco) in Neural Basal Medium (NBA, 10888-022, Gibco) at 37 °C, 5% CO_2. Following a 40 min incubation time, the medium containing collagenase was removed, and the ganglia were washed using Hank's buffered salt solution (H9269, Sigma). The ganglia were further enzymatically dissociated using 1 mg/mL trypsin (25300-054, Gibco) in the neural medium for 15 min at 37 °C, 5% CO_2. At the end of incubation, 50 mg/mL DNAse (D4513, Sigma) was added to the trypsin solution cell suspension to inhibit free DNA fragments, and the tissues were triturated by pipetting to obtain single cells. 30 min after incubation at 37 °C, 5% CO_2, the cell suspension was spun at 120g for 3 min and resuspended in neural medium supplemented with 10% fetal calf serum and 700 mg/mL trypsin inhibitor (T6522, Sigma) to inhibit enzymatic activity. To purify DRG neurons from the satellite cells and cell debris, the cell suspension was carefully put into 10%, 35%, and 60% percoll (P4937, Sigma) gradients and spun at 300g for 20 min. The total sensory neurons were collected from approximately 35% percoll layer, and the cell suspension was spun at 120 g for 3 min. The pellet was resuspended with a neural medium without antibiotics for viral transduction. Following that procedure, cells were seeded onto a petri dish which had been previously coated with poly-L-lysine (P6282, Sigma) (1.8 µg/cm^2, 2 hours at RT) and then laminin (L2020, Sigma) (40ng/mm^2, overnight at 37 °C). One day after incubation, cells were transduced with lentivirus, as described above.

High titer virus purification

HEK293T cells were transfected with helpers MDL, RSV-rev, VSVG (gifts from Didier Trono, Addgene plasmids #12251, #12253, and a gift from Arthur Nienhuis & Patrick Salmon, Addgene plasmid #35616,) together with transfer plasmids (pLenti-MP2-SypHer3s or pLenti-MP2-DsRed-stDCyD) with 1:4 plasmid:PEI ratio when reached to 70 % confluency. Lentivirus-containing medium was harvested at 72h of transfection followed by centrifugation at 3000 g for 3 min and filtered through 0.45 um PES filter (SLHP033RS, Millipore). Lentiviral particles were purified via centrifugation at 10,000g for 4h at 4°C on

a 20% sucrose cushion and further concentrated with the same centrifugation parameters in ice-cold 1x PBS.

For the generation of high titer ASAP2s viral particles, AAV293 cells at 70% confluency were transfected with pAAV-hSyn-ASAP2s, pAdDeltaF6, and pAAV2/1 (a gift from Francois St-Pierre, and gifts from James M. Wilson, plasmids were purchased from Addgene plasmids #112867, #112867, #101276) with 1:4 plasmid:PEI ratio. Cells were collected with a cell scraper 72h after transfection and pelleted by centrifugation at 300g for 5 min. Cell pellets were resuspended in lysis buffer (150 mM NaCl, 20 mM Tris, 1mM $MgCl_2$, pH:8) and lysed following a 3x freeze-thaw cycle and subsequent sonication and Benzonase (E8263, Sigma) treatment for 45 min at 37°C. Cell debris was removed with centrifugation at 300g for 20 min at 4°C. AAV containing supernatant was ultracentrifuged at 220.000g through Iodixanol (D1556, Sigma) gradient (60%, 40%, 25%, 17%) for further purification for 2h. AAV particles were collected from the interface between gradients of 40%-60%. Iodixanol was removed, and AAV particles were concentrated in a storage buffer (1x PBS, 5% D-sorbitol, 200 mM NaCl) using 100K columns (UFC910024, Millipore) with 3x centrifugation at 4°C and 300g for 30 min.

Live cell imaging

Widefield imaging experiments were performed on a Zeiss Axio Observer.Z1/7 (Carl Zeiss AG, Oberkochen, Germany) equipped with an LED light source Colibri 7 (423/44 nm, 469/38 nm, 555/30), Plan-Apochromat 20×/0.8 dry objective, Plan-Apochromat 40×/1.4 oil immersion objective, a monochrome CCD camera Axiocam 503. A custom-made pump-driven perfusion system was used to administrate and withdraw substrates to cells placed in a metal perfusion chamber (NGFI, Graz, Austria). SypHer signals were imaged by alternately exciting cells using a motorized dual-filter wheel equipped with beam splitters (FT455 (for SypHer low, F420) and FT495 (for SypHer high, F490)). Emissions were alternately collected using a bandpass filter (BP 525/50). ASAP2s was excited at 477nm and emission was collected at 525/50 nm. DsRed-stDyCD emission was collected using the filter combinations FT570 (BS) and emission filter 605/70. During live-imaging data acquisition was performed using Zen Blue 3.1 Pro software (Carl Zeiss AG, Oberkochen, Germany). *Confocal imaging* was performed using a laser scanning confocal microscope LSM 800 (Zeiss, Germany) equipped with a Plan-Apochromat 40x/1.3 DIC (UV) VIS-IS oil immersion objective. SypHer biosensors were excited with a 488 nm and 405 nm laser, and emissions were collected using a 509 nm filter system. SypHer signals were acquired with an A GaAsP-PMT detector and 400–565 nm filter using a Multialkali-PMT detector. DsRed-stDCyD constructs were excited using a 561 nm laser, and the emission wavelength was captured between 616 and 700 nm. The digital detector gain for all channels was set at 1; detector gain was applied between 500 and 1000 V. Laser intensities were set between 0.90% and 0.95%, and the pinhole was set between 29 and 32 μm according to the expression level of the fluorescent proteins. Bright-field mode was imaged using a photodiode detector. Zen Blue 3.1 software (Zeiss, Germany) was used to determine regions of interest.

Statistical Analysis

Image analysis was performed using GraphPad Prism software (GraphPad Software, San Diego, CA, USA). All experiments were repeated at least in triplicates, and the exact number of experiments is given as' N', and the total number of cells imaged is indicated as' n.' in the figure legends. For instance: 3/32 indicates N = 3 (triplicate cultures) and n = 32 (number of cells imaged in this experiment). Statistical comparison of the two groups was evaluated using a two-tailed Student's t-test.

Theoretical Calculations

To estimate the percentage of increase in concentrations of the enzymatic reaction (by)products when the pH drops from 7 to 6, we assumed that the HEK293T cell volume is 5×10^{-12} L and initial concentrations of the reaction byproducts (i.e., chloride ion, pyruvate, and ammonia) are as listed in Table S1. Using equation 1, the initial and final concentrations of hydronium ions were calculated based on the pH values. $[H^+] = 10^{-pH}$ (1)To calculate the number of moles from the concentration, the concentration was multiplied by the cell volume. As the enzymatic reaction (by)products are generated equimolarly, an increase in the moles of hydronium ions was used to measure the final moles of other (by)products. The final concentrations of the (by)products were then calculated by dividing the final moles by the cell volume. Finally, the percentage of alteration in concentration was determined using equation 2, which compares the final and initial concentrations of the (by)products.

Percentage of Alteration = [(Final concentration - Initial concentration) / Initial concentration] x 100% (2)

Ethics

The Istanbul Medipol University's ethical committee approved animal experimentation. Maximum care was paid with total commitment to the 3R principles to use the minimum number of animals necessary for the research aims and to minimize suffering throughout the study. All procedures have complied with European Council Directive 2010/63/EU. Breeding colonies of wild-type male C57BL/6 mice were obtained from The Jackson Laboratory and bred in the Istanbul Medipol University Experimental Animal Facility.

Table S1

Name	Initial concentration (M)	Final concentration (M)	İnitial moles	Final moles	Percentage of concentration alterations
**Hydronium Ion**	10^{-7}	10^{-6}	$5*10^{-19}$	$5*10^{-18}$	900%
**Chloride ion**	$5*10^{-3}$	$5.0009*10^{-3}$	$2.5*10^{-14}$	$2.50045*10^{-14}$	0.018%
**Pyruvate**	$4*10^{-5}$	$4.1*10^{-5}$	$2*10^{-16}$	$2.045*10^{-16}$	2.5%
**Ammonia (blood)**	$2*10^{-5}$	$2.09*10^{-5}$	10^{-16}	$1.045*10^{-16}$	5%

Table S1 | Theoretical calculations of %-increase in the concentration of ions and molecules produced as (by)products of stDCyD enzyme activity upon treatment with βCDA when the pH decreases by one order of magnitude (i.e., from 7 to 6). The initial concentrations are the rough average level of ions/molecules in the human body (whether a single cell or tissue) taken from literature. The (by)products are considered to be generated equimolarly, and the average volume of cells (HEK 293T) is assumed to be $5*10^{-12}$ L.

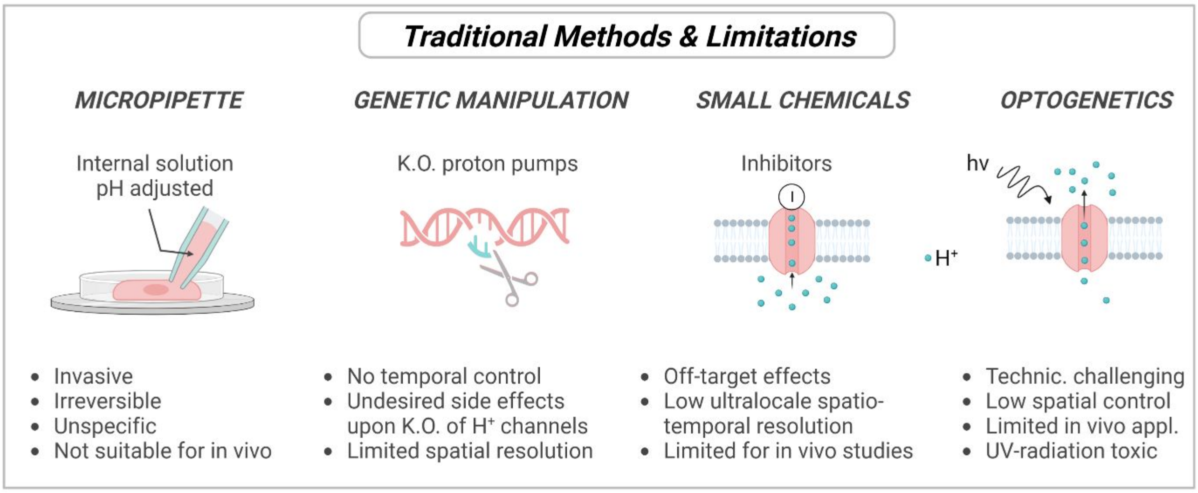

Figure S1 | Typical techniques to change the intracellular pH (pHi) of intact cells fall into the following categories: (*First panel*) In clamped single cells, pHi can be altered using the perfused-pipette approach. However, perforating cells is invasive, limited to a single cell, technically tricky, and ineffective for animal research studying the impact of pH perturbations in a cell population or tissue. (*Second and third panels*) There are several primary groups of proton pumps, each with a unique energy source, polypeptide composition, and evolutionary history, which undermines the controlled regulation and expression levels, thus the functionality in cells and tissues. Both gene silencing and chemical inhibition do not permit temporal and spatial control of acidification. In addition, proton transporters also co-transport other ions, therefore, inhibition of these transporters can have detrimental effects on the ion homeostasis. (*Fourth panel*) Optogenetic outward proton pumps help elicit robust and physiological pHi increases over time, yet with limited spatial resolution. Overall, the major drawbacks of these approaches are: that they do not permit ultra-local manipulation on an organellar level, and they are poorly combinable with simultaneous detection methods such as genetically encoded biosensors for live-cell imaging.

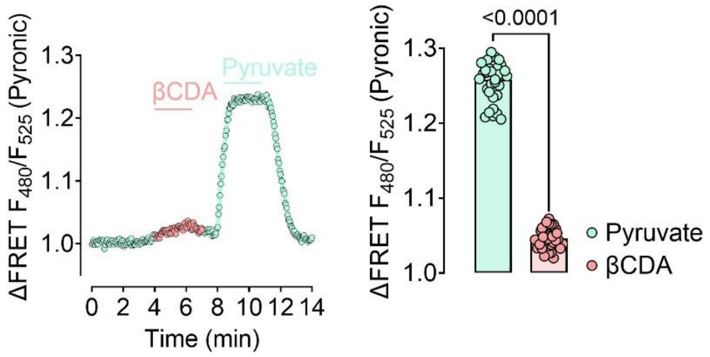

Figure S2 | Visualizing intracellular pyruvate, a byproduct of the pH-Control activity. (*Left*) Real-time traces of the FRET-biosensor Pyronic[2] in response to 11 mM βCDA and 1 mM pyruvate. (*Right*) Bars represent the maximum FRET response upon administration of extracellular pyruvate (n=5/34) and βCDA (n=5/34). Student's t-test has been applied.

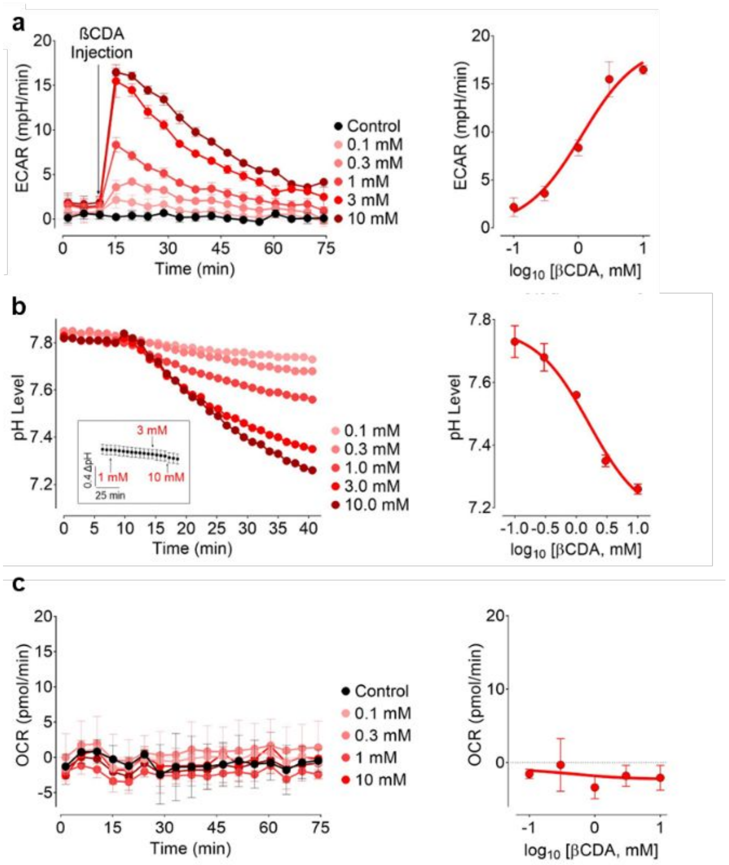

Figure S3 | In vitro characterization of purified Ds-Red-stDCyD using Seahorse XFe96 Analyzer. **a,** (Left) Real time traces of extracellular acidification rate (ECAR) using 100 nM purified DsRed-stDCyD with indicated concentrations of βCDA. The right panel shows the concentration-dependent correlation between βCDA and ECAR. **b,** (Left) Real time changes in pH upon administration of indicated concentrations of βCDA (legends) to Seahorse medium containing 100 nM purified DsRed-stDCyD. Inset shows the real time changes in pH in Seahorse medium without purified DsRed-stDCyD after constitutive injection of βCDA (arrows indicate the time and concentration). The right panel shows the concentration-dependent pH-change. **c,** (Left) Real time traces of oxygen consumption rate (OCR) after administration of the indicated concentrations of βCDA (concentrations are given in the legends). The right panel shows concentration-dependent OCR fluctuations. All the experiments are the average of technical triplicates and error bars are given as ±SD.

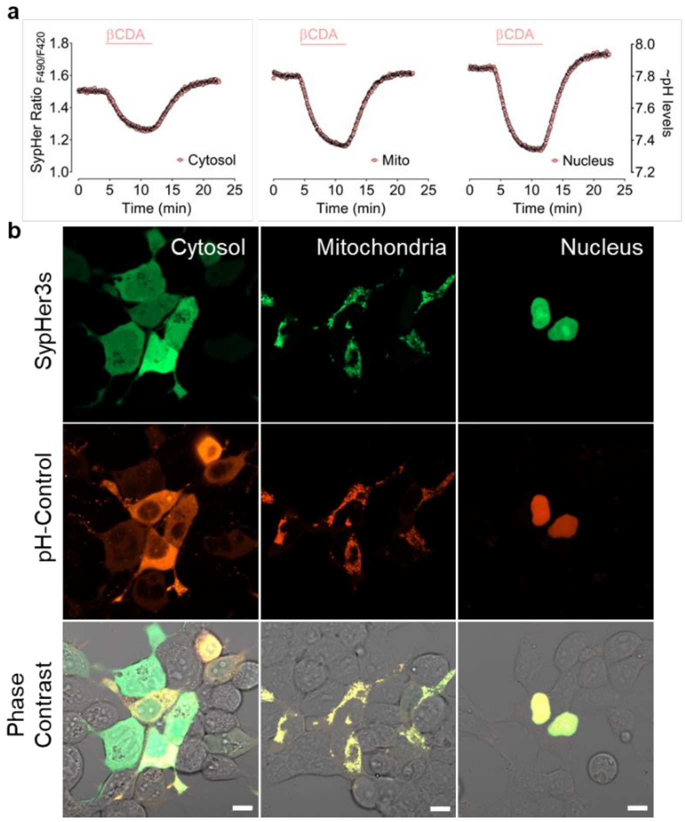

Figure S4 | Manipulating subcellular pH levels. a, Real-time traces of SypHer3s biosensor in three different cellular locales, including the cytosol (n=3/16), mitochondria (n=3/22), and nucleus (n=3/33) in response to 1 mM βCDA. **b,** Representative confocal images show the correct localization of the pH-sensitive biosensor SypHer3s (upper panel) and the pH-Control constructs (middle panel). Lower panels show merged fluorescence images with bright field images.

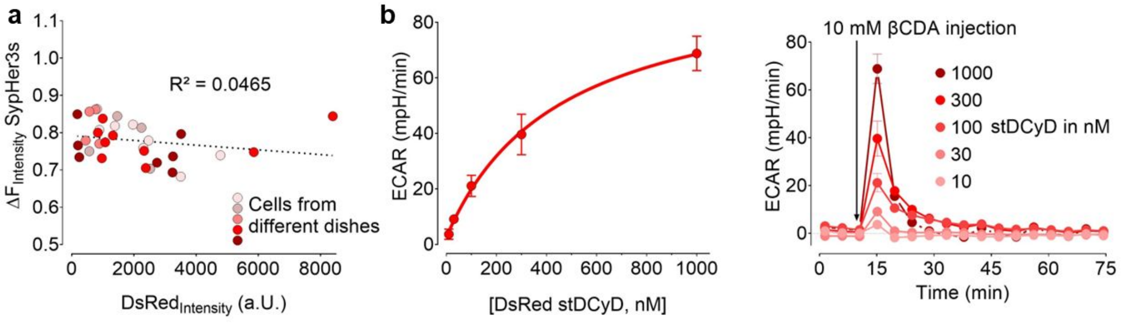

Figure S5 | Correlation between stDCyD concentration and its acidification capacity. a, Scattered dot plot shows the correlation between expression levels of stDCyD (measured by the fluorescent intensity of DsRed) and changes in SypHer3s ratio upon administration of 1mM βCDA. Experiments have been performed in HEK293 cells. 32 cells have been analyzed from 5 independent dishes as indicated with different colors. P= 0,2359. **b**, The left panel shows the correlation between various concentrations of purified DsRed-stDCyD (10, 30, 100, 300, and 1000 nM) and ECAR upon 10 mM βCDA. The right panel shows real-time changes in ECAR of the same experiment. All values are performed in triplicates, and error bars are given in ±SD.

MDNTEDVIKEFMQFKVRMEGSVNGHYFEIEGEGEGKPYEGTQTAKLQVTKGGPLPFAWDILSPQFQYGSKAYVKHPADIPD
YMKLSFPEGFTWERSMNFEDGGVVEVQQDSSLQDGTFIYKVKFKGVNFPADGPVMQKKTAGWEPSTEKLYPQDGVLKGEI
SHALKLKDGGHYTCDFKTVYKAKKPVQLPGNHYVDSKLDITNHNEDYTVVEQYEHAEARHSGSQVDATMPLHHLTRFPRLEF
IGAPTPLEYLPRLSDYLGREIYIKRDDVTPIAMGGNKLRKLEFLVADALREGADTLITAGAIQSNHVRQTAAVAAKLGLHCVALL
ENPIGTTAENYLTNGNRLLLDLFNTQIEMCDALTDPDAQLQTLATRIEAQGFRPYVIPVGGSSALGAMGYVESALEIAQQCEE
VVGLSSVVVASGSAGTHAGLAVGLEHLMPDVELIGVTVSRSVAEQKPKVIALQQAIAGQLALTATADIHLWDDYFAPGYGVP
NDAGMEAVKLLASLEGVLLDPV**F**TGKAMAGLIDGISQKRFNDDGPILFIHTGGAPALFAYHPHVDIIDIITG*

↑

Single mutation in stDCyD
(**Y**287**F**)

Figure S6 | Primary sequence of pH-Control. The red highlighted sequence represents the primary sequence of the red fluorescent protein DsRed, and the light grey highlighted sequence is the primary sequence of the *Salmonella Typhimurium*-derived enzyme termed stDCyD. Single mutation has been introduced to position 287 as indicated to yield a nonfunctional enzyme.

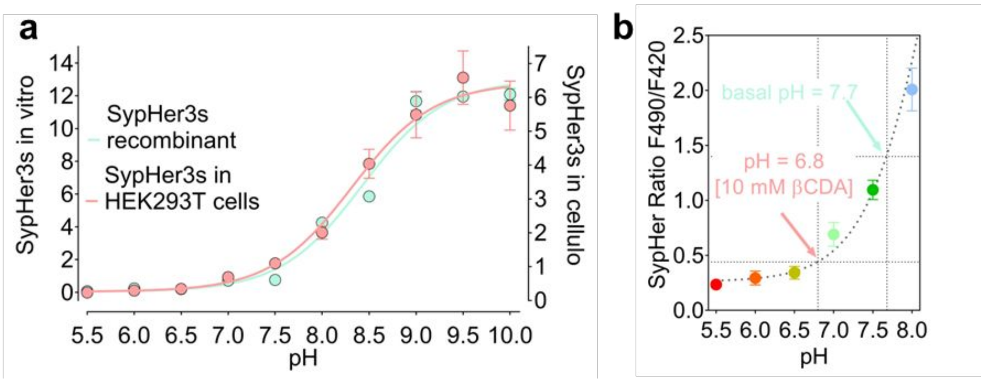

Figure S7 | Calibration of the pH biosensor SypHer3s. a, pH correlation curves show SypHer3s ratio values in response to different pH levels. Cyan curves and dots show purified recombinant proteins (each dot represents technical triplicates) in vitro, while the pink curves and dots show SypHer3s response in HEK293 cells (pH=5.5, n=4/59; pH=6.0, n=4/43; pH=6.5, n=4/49; pH=7.0, n=4/63; pH=7.5, n=4/47; pH=8.0, n=4/52; pH=8.5, n=4/49; pH=9.0, n=4/55; pH=9.5, n=4/35; pH=10.0, n=4/47). Cells were incubated with 5 µM digitonin, 0.7 µM monensin, and 0.7 µM nigericin for 12 minutes before imaging experiments. **b,** Close-up pH dependent SypHer3s ratio values between pH 5.5 and 8.0 is shown. Error bars are given in ±SD.

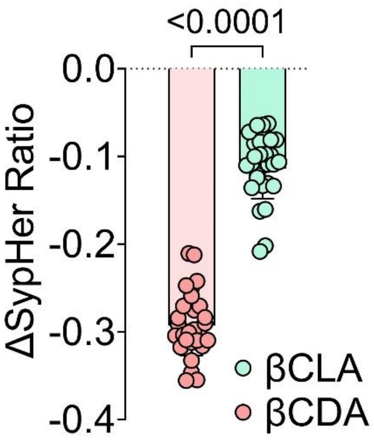

Figure S8 | Selectivity of stDCyD: Bars show the selectivity test of cells treated with 1mM βCDA (n=3/29) or 1mM βCLA (n=3/29). Student's t-test has been performed and error bars are given in ±SD.

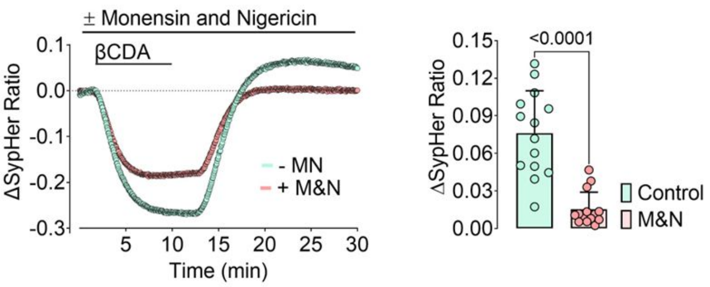

Figure S9 | overcorrection of intracellular pH levels following acute acidification. (*left*) Real-time traces of HEK293T cells expressing SypHer and pH-Control in response to 1 mM βCDA in the presence (pink curve and (*right*) bars, n=3/15) and absence (green curve and (*right*) bars n=3/13) 10 μM nigericin and 10 μM monensin that permeabilize the plasma membrane. Student's t-test has been applied.

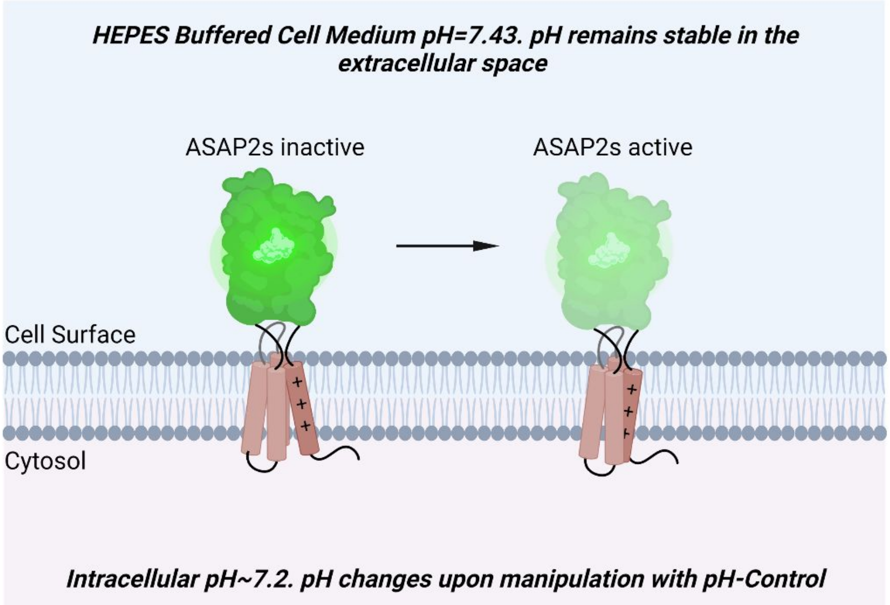

Figure S10 | Schematics of the voltage sensor ASAP2s. The cartoon shows the principle of ASAP2s located in the cell's outer membrane. Only intracellular changes in the voltage affect the brightness of the sensor. Thus, the effective change in pH inside the cells does not affect the circularly permuted green fluorescent protein, which is part of the biosensor and might be pH sensitive. Also, the pH buffering capacity in the extracellular imaging medium (10 mM HEPES), which is under continuous flow, stabilized the pH level outside the cell to pH=7.43 and prevent any pH-dependent (de)activation of the probe. Chemogenetic manipulation with pH-Control in the cell cytosol changes intracellular pH levels and modulates the membrane voltage of the cells yet cannot influence the cpGFP. Thus, pH-control mediated ASAP2s signals are less likely to be pH artifacts.

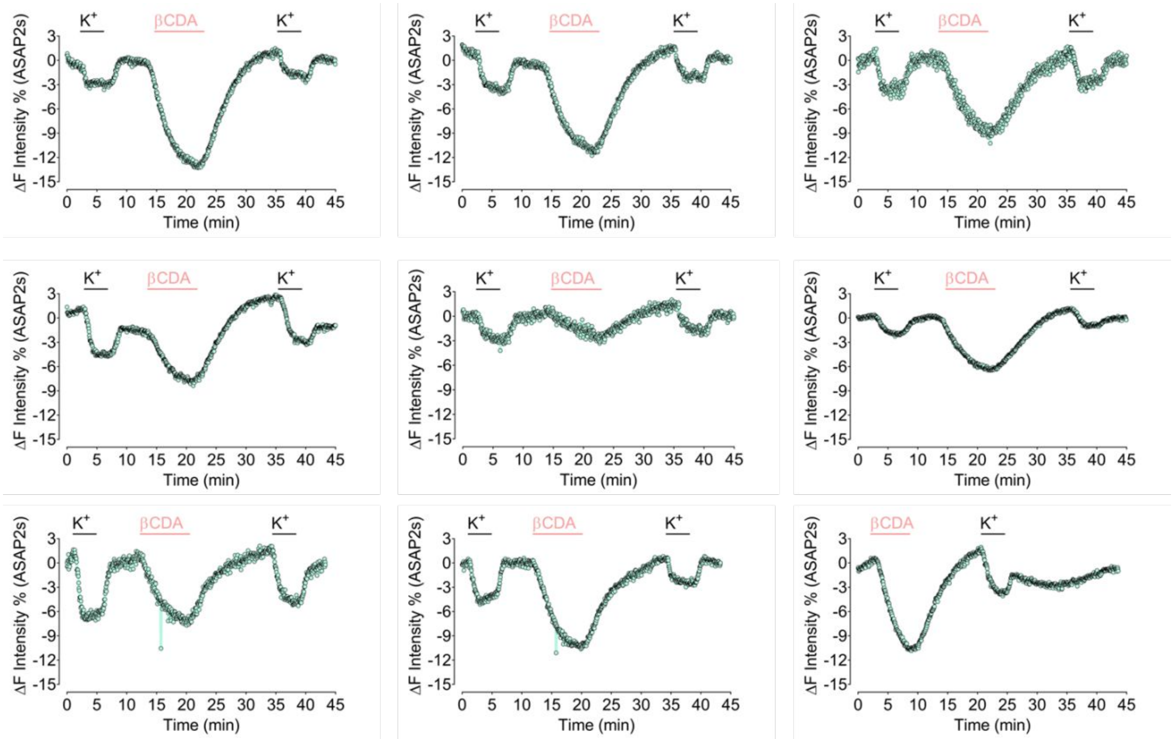

Figure S11 | pH-Control permits modulation of the membrane potential in primary neurons. Representative real-time traces of ASAP2s signals in primary DRG neurons co-expressing pH-Control and ASAP2s in response to 10 mM βCDA or high extracellular K^+ levels (50 mM) and low Na^+ levels (93 mM). Individual cell responses are from 4 independent experiments from two different batches of animals.

Supplementary Note 1: Acknowledgement of Variability in Basal pHi Values for HEK293T Cells. We acknowledge that our measured basal pHi values for HEK293T cells (pH= 7.7) may differ from some other published data that utilized other probes (e.g., BCECF)[3–5]. In this study, the pHi values reported are based on our calibration experiments of the SypHer3s biosensor, both *in cellulo* and *in vitro*. We emphasize that we used these values exclusively to determine and represent the efficacy of our tool in manipulating pHi levels. These calibration experiments were performed using a carefully controlled and well-characterized experimental system, and we believe our reported pHi values to be accurate within this context. Our findings indicate the potential utility of SypHer3s and pH-Control as reliable tools for visualizing and manipulating pHi in HEK293T cells, respectively, and we encourage further investigations to expand the application of these tools in other cell types and experimental settings.

Supplementary Table 2

Plasmid Name	Forward Primer	Reverse Primer	Templae Name
Untargeted DsRed-stDCyD	**BamHI-DsRed-stD-FWD**	**Apal-stop-Agel-stD-dsRed-REV**	pTwist CMV BetaGlobin
	ATAATAGGATCCTCTAGAGCCACCATGGACAACACCG	TATTATGGGCCCTTAACCGGTTATGATATCTATGATATCAACGTGGG	
mito-DsRed-stDCyD	**Mito-stD-BamHI-FWD**	**Mito-stD-Xbal-REV**	mito-mCherry-mDAAO[6]
	TATGGATCCGAATTCGCCACCATGTCTGTTC	ATATCTAGACTTGGCCCTGGGGACTG	
DsRed-stDCyD-NES	**Agel-stD-NES-FWD**	**stD-NES-Apal-REV**	pCS2+H7-NES (Addgene Plasmid #136467)
	TATACCGGTCTGCCCCCCCTGGAG	ATAGGGCCCGTGGCAACTTCCAG	
DsRed-stDCyD-NLS	**stD-Agel-NLS-FWD**	**stD-NLS-Apal-REV**	HyPer7 NLS[6]
	CAAGTTCTGCTTCTGACCGAGGGC	ATAGGGCCCTTCTAGAGGCTCGAG	
Untargeted SypHer	**BamHI-UN.SypHer-pLenti-FWD**	**Xbal-UN.SypHer-pLenti-REV**	Sypher3s (Addgene Plasmid #108118)
	TATGGATCCACTAGTGCCACCATGTCCGGAC	GATATCTAGATTAACGCGTCTCGAGAACCGCCTGTTTTAAAAC	
mito-SypHer	**Xbal-SypHer-mito-pLenti-FWD**	**Apal-SypHer-mito-pLenti-REV**	mito-mCherry-mDAAO[6]
	TATTCTAGAGCCACCATGTCCGGACCGCTG	GCAATAGGGCCCTTAAACCGCCTGTTTTAAAACTTTATCG	
SypHer-NES	**MluI-SypHer-NES-pLenti-FWD**	**Xbal-SypHer-NES-pLenti-REV**	pCS2+H7-NES (Addgene Plasmid #136467)
	TATACGCGTCTGCCCCCCCTGGAG	ATATCTAGAGTGGCAACTTCCAGGGCCAG	
SypHer-NLS	**MluI-SypHer-NLS-pLenti-FWD**	**stD-NLS-Apal-REV**	HyPer7 NLS[6]
	GTATACGCGTACCGGTGATCCAAAAAAG	ATAGGGCCCTTCTAGAGGCTCGAG	
dsRED-Y287F stDCyD	**SalI-stDcyD-For**	**Apal-stop-stDcyD-Rev**	pTwist CMV BetaGlobin
	CAGGTCGACGCCACCATG	TATCTGTACAGGGCCCTATGAATTC	
	**stDcyD-Y287F-For**	**stDcyD-Y287F-Rev**	
	CGATCCAGTTTTCACTGGGAAGG	GCCTTCCCAGTGAAAACTGGATCG	

References

(1) Bharath, S. R.; Bisht, S.; Harijan, R. K.; Savithri, H. S.; Murthy, M. R. N. Structural and Mutational Studies on Substrate Specificity and Catalysis of Salmonella Typhimurium D-Cysteine Desulfhydrase. *PLOS ONE* **2012**, *7* (5), e36267. https://doi.org/10.1371/journal.pone.0036267.

(2) Martín, A. S.; Ceballo, S.; Baeza-Lehnert, F.; Lerchundi, R.; Valdebenito, R.; Contreras-Baeza, Y.; Alegría, K.; Barros, L. F. Imaging Mitochondrial Flux in Single Cells with a FRET Sensor for Pyruvate. *PLOS ONE* **2014**, *9* (1), e85780. https://doi.org/10.1371/journal.pone.0085780.

(3) Nadtochiy, S. M.; Schafer, X.; Fu, D.; Nehrke, K.; Munger, J.; Brookes, P. S. Acidic PH Is a Metabolic Switch for 2-Hydroxyglutarate Generation and Signaling*. *Journal of Biological Chemistry* **2016**, *291* (38), 20188–20197. https://doi.org/10.1074/jbc.M116.738799.

(4) Ruminot, I.; Gutiérrez, R.; Peña-Münzenmayer, G.; Añazco, C.; Sotelo-Hitschfeld, T.; Lerchundi, R.; Niemeyer, M. I.; Shull, G. E.; Barros, L. F. NBCe1 Mediates the Acute Stimulation of Astrocytic Glycolysis by Extracellular K+. *J. Neurosci.* **2011**, *31* (40), 14264–14271. https://doi.org/10.1523/JNEUROSCI.2310-11.2011.

(5) Schuhmann, K.; Voelker, C.; Höfer, G. F.; Pflügelmeier, H.; Klugbauer, N.; Hofmann, F.; Romanin, C.; Groschner, K. Essential Role of the Beta Subunit in Modulation of C-Class L-Type Ca2+ Channels by Intracellular PH. *FEBS Letters* **1997**, *408* (1), 75–80. https://doi.org/10.1016/S0014-5793(97)00391-8.

(6) Erdogan, Y. C.; Altun, H. Y.; Secilmis, M.; Ata, B. N.; Sevimli, G.; Cokluk, Z.; Zaki, A. G.; Sezen, S.; Akgul Caglar, T.; Sevgen, İ.; Steinhorn, B.; Ai, H.; Öztürk, G.; Belousov, V. V.; Michel, T.; Eroglu, E. Complexities of the Chemogenetic Toolkit: Differential MDAAO Activation by d-Amino Substrates and Subcellular Targeting. *Free Radical Biology and Medicine* **2021**, *177*, 132–142. https://doi.org/10.1016/j.freeradbiomed.2021.10.023.
